# Tuning a bi-enzymatic cascade reaction in *Escherichia coli* to facilitate NADPH regeneration for ε-caprolactone production

**DOI:** 10.1186/s40643-021-00370-w

**Published:** 2021-04-22

**Authors:** Jinghui Xiong, Hefeng Chen, Ran Liu, Hao Yu, Min Zhuo, Ting Zhou, Shuang Li

**Affiliations:** 1 School of Biology and Biological Engineering, South China University of Technology, Higher Education Mega Center, Guangzhou, 510006 China; 2 School of Chemistry and Chemical Engineering, South China University of Technology, Guangzhou, 510006 China

**Keywords:** Whole-cell biocatalysis, RBS design, NADPH regeneration, Cyclohexanol, ε-caprolactone

## Abstract

ε-Caprolactone is a monomer of poly(ε-caprolactone) which has been widely used in tissue engineering due to its biodegradability and biocompatibility. To meet the massive demand for this monomer, an efficient whole-cell biocatalytic approach was constructed to boost the ε-caprolactone production using cyclohexanol as substrate. Combining an alcohol dehydrogenase (ADH) with a cyclohexanone monooxygenase (CHMO) in *Escherichia coli*, a self-sufficient NADPH-cofactor regeneration system was obtained. Furthermore, some improved variants with the better substrate tolerance and higher catalytic ability to ε-caprolactone production were designed by regulating the ribosome binding sites. The best mutant strain exhibited an ε-caprolactone yield of 0.80 mol/mol using 60 mM cyclohexanol as substrate, while the starting strain only got a conversion of 0.38 mol/mol when 20 mM cyclohexanol was supplemented. The engineered whole-cell biocatalyst was used in four sequential batches to achieve a production of 126 mM ε-caprolactone with a high molar yield of 0.78 mol/mol. 
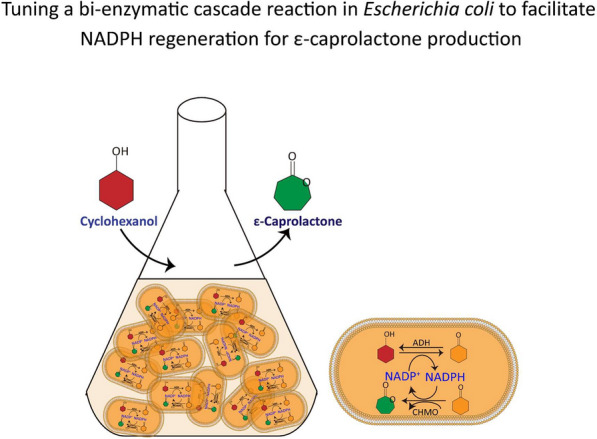

## Introduction

As people face the global changes in energy, resources, and the environment, biocatalysis attracts great attentions in chemical, pharmaceutical and energy industries because of its high activity, selectivity, specificity and low energy requirements. Oxidoreductases are one class of the most important enzymes (~ 25% of all enzymes) responsible for the inter-molecule electron transfer (Hollmann et al. 2011). However, many enzymatic redox applications are limited by the dependence on cofactors as hydrogen source, such as nicotinamide adenine dinucleotide (NADH) and its phosphorylated form (nicotinamide adenine dinucleotide phosphate, NADPH). Considering the high cost, stoichiometric usage and instability of NAD(P)H, an effective regeneration system is essential for the industrial implementation of oxidoreductases. It has been accepted as a rule of thumb that less than 0.1 mol% of the cofactor used appears economically (Hollmann et al. 2011).

Many critical reviews focused on the cofactor NAD(P)H regeneration have been documented by researchers (Quinto et al. 2014; Spaans et al. 2015; Wang et al. 2017a, b). In general, the regeneration of cofactor NAD(P)H can be conducted via enzymatic, chemical, photocatalytic and electrochemical approaches. Using inorganic salts with high redox potential or cofactor analogues, the NAD(P)H could be regenerated chemically (Wu et al. 2013). However, it has not been widely used for its inherent issues, such as low transformation efficiency, enzyme deactivation, waste generated, etc. Although photocatalytic and electrochemical methods attracted many attentions in recent years, more further studies and investigations are needed for industrial application due to its poor efficiency, bad compatibility and low selectivity (Hildebrand et al. 2008; Weckbecker et al. 2010). So far, only the enzymatic regeneration method has been demonstrated to be feasible and applied on industrial scale for its excellent compatibility with the target biocatalytic process (Sun et al. 2017; Xu et al. 2019; Zhang et al. 2019). In particular, the combination of redox enzymes (Wu et al. 2013), such as formate dehydrogenase and glucose dehydrogenase, in a linear cascade fashion has received increasing attentions because it does not need isolating intermediates.

**Fig. 1:**
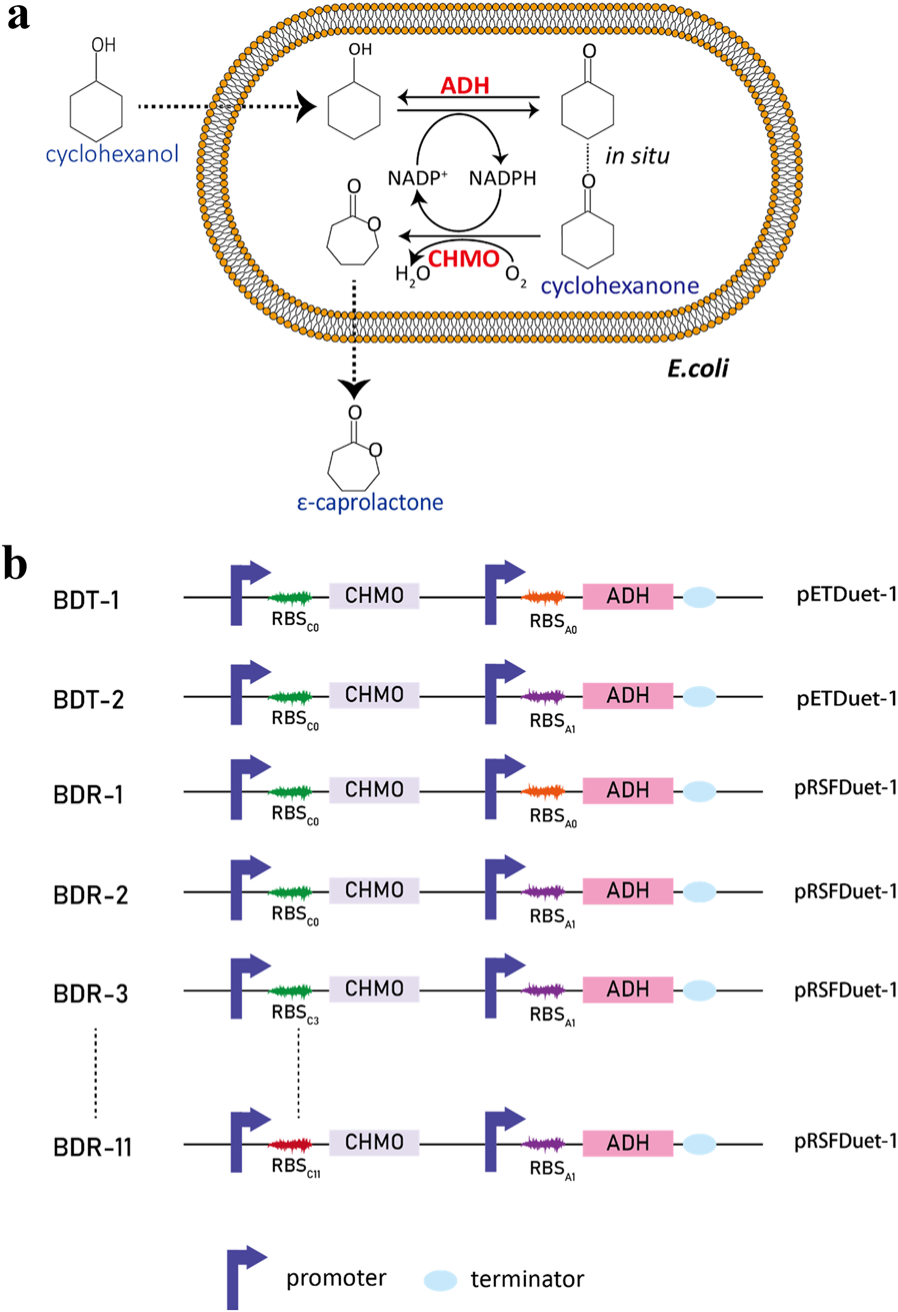
Bi-enzyme catalysis for ε-caprolactone production from cyclohexanol. **a** The schematic of a cascade reaction based on whole-cell biocatalysis system. Cyclohexanol is enzymatically converted into cyclohexanone by alcohol dehydrogenase (ADH) with NADPH generation, which is oxidized into ε-caprolactone by cyclohexanone monooxygenase (CHMO) with NADPH consumption. **b** Expression vector constructions to regulate the protein expression through RBS sequence replacements

ε-Caprolactone, with global annual production of multi-kilotons, is an important non-toxic compound used as a monomer for biodegradable, thermoplastic and elastomeric polymer synthesis(Pathak and Navneet 2017). In industry, ε-caprolactone is synthesized by chemical Baeyer–Villiger reaction using peroxycarboxylic acids as oxidant (ten Brink et al. 2004). In the early 1990s, Willetts et al. first reported an enzymatic approach to the lactone synthesis in a linear cascade fashion in vitro (Willetts et al. 1991). Nowadays, this cascade method has been applied to the ε-caprolactone biosynthesis, in which the oxidation starts from a readily available compound cyclohexanol by an alcohol dehydrogenase (ADH) and further undergoes the oxidation of cyclohexanone by a cyclohexanone monooxygenase (CHMO) (Mallin et al. 2013; Ménil et al. 2019; Scherkus et al. 2016; Schmidt et al. 2015b; Staudt et al. 2013; Xu et al. 2019). In this cascade reaction, the consumption of cyclohexanol to cyclohexanone catalyzed by ADH was accompanied by the production of NADPH, and the production of ε-caprolactone from cyclohexanone by CHMO was also accompanied by the consumption of NADPH (Fig. 1a). However, it is still debatable whether the catalysts are isolated enzymes, acellular extracts, or whole cells. From a practical point of view, whole cells containing all the enzymes in the same organism are preferred since external cofactor regeneration is not necessary. Moreover, tedious and high-cost enzyme purification processes are no longer required (France et al. 2017). However, it is still challenging to achieve satisfactory results in controlling the enzyme ratio in the cascade reaction system (Gandomkar et al. 2019; Ménil et al. 2019; Milker et al. 2017; Scherkus et al. 2017; Xu et al. 2019).

Herein, we systematically designed and constructed several mutants through engineering ribosome binding site (RBS) to achieve cofactor self-sufficient for ε-caprolactone production in *E. coli*. As a result, an optimized strain dramatically increased the ε-caprolactone molar yield and substrate tolerance. Finally, a sequential batch reaction was applied to further increase the production titer through the whole-cell biocatalytic approach.

## Material and methods

### Chemicals, strains and culture conditions

Cyclohexanol, cyclohexanone and ε-caprolactone were purchased from Sangon Biotech (Shanghai, China). The restriction enzymes and T4 DNA ligase were obtained from Thermo Fisher Scientific (Pittsburgh, PA, USA). DNA Polymerase of PrimeSTAR HS was from *TaKaRa* (Dalian, China). Plasmids of pETDuet-1 and pRSFDuet-1 (Novagen) were used for co-expression of the two genes.

*E. coli* DH5α was used for expression vector construction and plasmid maintenance. The strain *E. coli* BL21(DE3) was used as host for all expression experiments. *E. coli* cells were cultured in Luria–Bertani (LB) medium containing 10 g/L peptone, 10 g/L sodium chloride, and 5 g/L yeast extract with appropriate antibiotics (50 μg/mL kanamycin or 100 μg/mL ampicillin).

### Plasmid construction

The *CHMO* gene with quadruple mutant (GenBank Accession No. BAA86293.1) (Schmidt et al. 2015a) encoding cyclohexanone monooxygenase and the *ADH* gene (GenBank Accession no. AY267012.1) encoding alcohol dehydrogenase were both synthesized and cloned into pUC57 by Sangon Biotech (Shanghai, China) after codon optimization for expression in *E. coli*.

The *CHMO* and *ADH* were amplified by PCR using CHMO-f/CHMO-r and ADH-f/ADH-r primer pairs and integrated into the pETDuet-1 vector at the *Nco* I/*Pst* I and *Nde* I/*Xho* I sites, yielding pETD-CHMO and pETD-ADH, respectively. The dual-expression vectors of pET-C0A0 and pRSF-C0A0 were both constructed using the similar restriction enzyme digestion and ligation methods. All the constructed vectors and recombinant strains are listed in the Additional file 1: Table S1, and the corresponding amplification primers are shown in Additional file 1: Table S2.

We designed a 12-variant RBS library to tune expression ratio of the bi-enzyme cascade across a 500-fold range according the RBS Calculator v2.0 model (Additional file 1: Table S3) (De Novo DNA 2018; Salis et al. 2009). For RBS sequence change, a recombination-based cloning strategy was carried out using the ClonExpress II One Step Cloning Kit (Vazyme, Nanjing, China). All clones were sequenced for verification by Sangon Biotech (China).

### ADH and CHMO expression

Seed cultures were prepared by the inoculation of a single colony into 10 mL LB medium supplemented with appropriate antibiotics and incubated at 37 ℃, 220 rpm overnight. The overnight cultures were transferred into fresh LB medium (2%, v/v) and cultivated in 500-mL flasks. At a range of the cell density (OD_600_) between 0.5 and 0.7, isopropyl β-d-1-thiogalactopyranoside (IPTG) was added to a final concentration of 0.5 mM for the induction of enzymes expression. After incubating at 30 ℃ with 180 rpm in an orbital shaker for 7 h, the recombinant *E. coli* cells were harvested by centrifugation at 3050 rpm for 30 min and subjected to the activity evaluation.

Sodium dodecyl sulfate–polyacrylamide gel electrophoresis (SDS-PAGE) was used to detect the expression level.

### Whole-cell enzyme activity assay

The oxidation of NADPH assay was applied to determine the activities of ADH and CHMO in *E. coli* cell lysates (Schmidt et al. 2015b). Normalized 10 OD_600_ cells was suspended in 50 mM sodium phosphate buffer (pH 7.5) to a final volume of 1 mL and lysed by sonication. The supernatants were collected by centrifugation at 12,000 rpm at 4 ℃ for 15 min. Catalytic reactions were conducted in a final volume of 200-μL system containing 187 μL 50 mM sodium phosphate buffer (pH 7.5), 1 μL of 50 mM NADPH stock solution, and 10 μL crude cell lysate with 2 μL substrate stock. 1 mM acetophenone or 1 mM thioanisole were used as the substrate for measurements of ADH or CHMO activity, respectively. After 2 min of incubation at room temperature, the initial consumption rates of NADPH were calculated and normalized as enzyme activities in crude cell lysate in U/mL (μmol/min/mL).

### ε-Caprolactone production through whole-cell catalysis

The collected cell pellets were washed with a 20 mM Tris–Cl buffer (pH 7.5) with 1% NaCl and 1% dimethyl sulfoxide (DMSO) to a wet cell weight (WCW) of 100 g/L. This cell suspension was chilled in ice-water bath for 30 min and centrifuged again at 1050 g, 4 °C for 25 min. Cell pellets were collected and resuspended in 20 mM Tris–Cl buffer (pH 7.5) containing 1% NaCl yielding 10 g_wcw_/L resting cell suspensions as whole-cell biocatalyst. The biocatalysis were carried out in 50 mL Erlenmeyer flasks containing 10 mL of the whole-cell biocatalyst supplemented with cyclohexanol as substrate at different concentrations. The flasks were sealed with Parafilm® and incubated at 25 ℃, 120 rpm for 16 h.

Sequential fed-batch catalysis was conducted in 250-mL flasks with reaction volume of 50 mL. The recombinant cells were cultured, induced, collected and pretreated as described above. The initial reaction mixture, consisting of 20 mM Tris–HCl buffer (pH 7.5) with 1% NaCl, resting cells (10 g_wcw_/L) and 40 mM cyclohexanol, was incubated at 25 °C and 120 rpm for 16 h. Subsequently, cyclohexanol was fed to the reactor at a final concentration of 40 mM at 16, 32 and 48 h.

Samples of 0.5 mL were taken and stored at − 20 °C for the determination of cyclohexanol, cyclohexanone, ε-caprolactone. All catalytic experiments were performed in triplicates.

### Analysis of cyclohexanol, cyclohexanone, ε-caprolactone

Aliquots of the reaction mixture (500 μL) were taken and extracted using equal volume of ethyl acetate containing 2 mM acetophenone as internal standard. The organic phase was collected after vigorous vortex for 15 min and centrifugation at 12,000 rpm for 10 min. The upper organic layer of the mixture was appropriately diluted with ethyl acetate, and then filtered with filter membrane (0.22 μm) into the sample vials for analysis.

Gas chromatography (GC) analyses were performed using a Hewlett-Packard 7890 Gas Chromatography (Agilent) equipped with a HP-5 column (crosslinked 5% Ph-Me Siloxane; 30 m × 0.32 mm × 0.25 μm) and a hydrogen flame-ionization detector (FID). The temperatures of injector and detector were set as 250 ℃ and 280 ℃, respectively. 1 μL sample was injected with a split ratio of 10:1 at a rate of 2 mL/min in constant flow mode (33.093 cm/s linear velocity). Samples were run using the following program: the initial GC oven temperature was set to 37 °C, then increased to 160 ℃ with a ramp rate of 10 ℃ per min, and the temperature finally was increased to 220 ℃ with a ramp rate of 20 ℃ per min. The concentrations of substrates and products were determined using internal calibration curves.

Chromatography coupled to mass spectrometry (GC − MS) analyses were performed on a GC/MS-HP7890 (Agilent) gas chromatography system equipped with a 5975C series mass selective detector (MSD) and a HP-5 column (crosslinked 5% Ph-Me Siloxane; 30 m × 0.32 mm × 0.25 μm). The same GC oven temperature programs were used as described above for GC detection. MS data were recorded at 70 eV (EI), *m/z* (rel. intensity in %) as TIC, total ion current. The sample was analyzed in a mass/charge (*m/z*) range of 40–200. Compounds in the samples were determined by comparing retention time and mass spectrometry GC/MS spectra to the commercially available chemical standards and mass spectrometry data from the NIST Standard Reference database.

The statistical analysis multiple *t* tests were performed by GraphPad Prism 8.0. The false discovery rate (FDR) approach using two-stage step-up method of Benjamini, Krieger and Yekutieli was chosen for analysis. Significant difference was considered when *p* value < 0.05.

### Detection of NADPH/NADP^+^ ratio in cells

The ratio of NADPH/NADP^+^ was detected using Coenzyme II NADP(H) Assay Kit (Comin, Suzhou, China). Samples at 0 h and 16 h during the whole-cell catalysis using 60 mM cyclohexanol as substrate were taken for detection. All experiments were performed in triplicates.

## Results and discussion

### ADH and CHMO expression and enzyme activity determination

To balance the NADP(H) regeneration and intermediate consumption during the ε-caprolactone bi-enzymatic synthesis, the ratio of ADH and CHMO is very important for their different specific activities and expression levels. A mutant of CHMO (C376L/M400I/T415C/A463C) with better stability at high temperatures from *Acinetobacter calcoaceticus* (Schmidt et al. 2015a) and ADH from *Lactobacillus kefir* were cloned into the MSC1 (RBS_C0_) and MSC2 (RBS_A0_) in pETDuet-1, yielding pETD-CHMO and pETD-ADH, respectively (Fig. 1, Additional file 1: Table S1). When vectors were transformed into *E. coli* BL21(DE3), the recombinant cells were cultured and induced by 0.5 mM IPTG for the expression of cyclohexanone monooxygenase and alcohol dehydrogenase. After incubation for 7 h, specific activities of ADH and CHMO in crude cell lysates were determined as 1.66 and 0.1 U/mL in BDT-1, 2.9 and 0.2 U/mL in BDR-1, respectively. Apparently, there was a wide gap in the crude activities between ADH and CHMO.

### ADH expression level alteration

To balance the catalytic efficiency for ADH and CHMO in a co-expression vector, a reduction of the ADH expression level would be preferred. In the most cases, the translation initiation is the rate-limiting step in bacterial protein expression. The ribosome binding site (RBS) and some other regulatory RNA sequences are thought as effective elements for the control of translation initiation (Salis et al. 2009). It is generally accepted that a purine-rich Shine–Dalgarno (SD) sequence similar to 5′-GGAGG-3′ located on RBS functions as a region with high affinity for the 30S subunit binding. Mutation at the SD sequence could severely reduce the expression level of the target protein in *E. coli* (Ban et al. 2000).

**Fig. 2:**
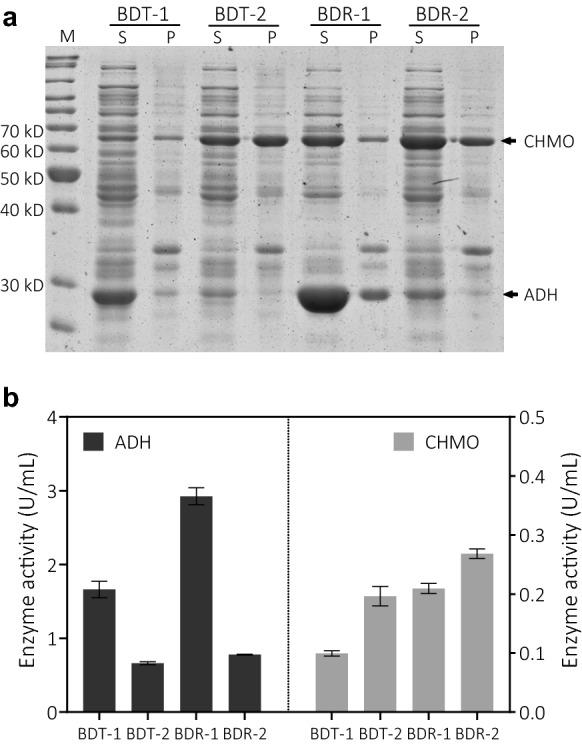
Expression levels of *E. coli* BL21(DE3) containing alcohol dehydrogenase (ADH) and cyclohexanone monooxygenase (CHMO) in the dual promoter vectors. **a** SDS-PAGE electropherogram. 10 OD_600_ Cells were collected by centrifugation and resuspended in 1 mL lysis buffer and subjected to sonication for cell lysis. *S* = supernatant fractions of cell lysates, *P* = precipitates of cell lysates, *M* = standard protein marker. The arrows indicate CHMO (62 kDa) and ADH (28 kDa), respectively. **b** Enzyme activity (U/mL) of ADH and CHMO for the four recombinant *E. coli* strains. Measurements were performed in three independent experiments

In this study, a mutation of G → C at the SD sequence on RBS_A0_ controlling ADH expression was introduced (from 5′-GGAGA-3′ to 5′-GCAGA-3′). The plasmids containing the CHMO and ADH expression cassettes based on pETDuet-1 and pRSFDuet-1 were constructed and transformed into *E. coli* BL21(DE3) cells separately, yielding BDT-1, BDT-2, BDR-1 and BDR-2 (Fig. 1). Different expression vectors containing variable SD sequences had little effects on the cell growth (Additional file 1: Table. S4). However, the specific activities in the crude cell lysates and SDS-PAGE picture clearly demonstrated the changes in the ADH and CHMO expression levels (Fig. 2a). Changes of purine to cytosine on SD sequence on RBS_A0_ caused the ADH activity reduction from 1.66 U/mL (BDT-1) to 0.66 U/mL (BDT-2) in pETDuet-1. On the contrary, an increase in CHMO activity based on pETDuet-1 was observed from 0.10 U/mL to 0.19 U/mL. When using pRSFDuet-1 as the construction backbone, similar variation tendencies of expression levels and specific activities for ADH and CHMO were recorded. To be noted, whatever the SD sequence is, the expression levels of CHMO and ADH on pRSFDuet-1 were all higher than those on pETDuet-1. The copy number of pETDuet-1 plasmid containing ColE1 replicon was less than that of pRSFDuet-1 vector with RSF1030 replicon, which might lead to much lower expression levels of targets on pETDuet-1 than pRSFDuet-1 (Tolia and Joshua-Tor 2006).

**Fig. 3:**
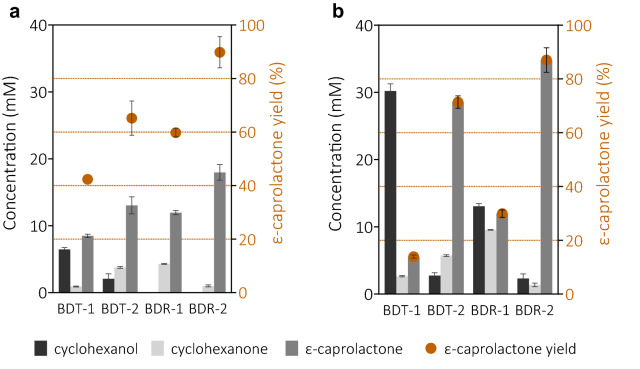
Whole-cell biocatalysis of 20 mM (**a**) and 40 mM (**b**) cyclohexanol into ε-caprolactone in 50 mL Erlenmeyer flasks containing 10 mL of cell catalysts (10 g/L WCW). Catalysis was carried out at 25 ℃ for 16 h. The data were expressed as mean values with standard deviations calculated from triplicate independent experiments

To evaluate the performance of the four bi-enzymatic cascades, whole-cell bioconversion of cyclohexanol to ε-caprolactone was carried out feeding with 20 mM or 40 mM of substrate cyclohexanol (Fig. 3 and Additional file 1: Fig. S1). For the strains of BDT-1 and BDR-1 containing the native SD sequence, only 8.5 and 11.9 mM of ε-caprolactone were produced when 20 mM of substrate cyclohexanol was fed. When the site mutation was introduced, 20 mM of cyclohexanol was totally converted with an ε-caprolactone yield of 65.2 and 87.0% for BDT-2 and BDR-2, respectively. However, a small amount of cyclohexanone was detected in the reaction mixture. When the initial substrate concentration was increased to 40 mM, the differences in bi-enzymatic cascade reactions were even more pronounced (Fig. 3b). The performance of strain of BDT-1 and BDR-1 was not good enough, as the ε-caprolactone yields were dramatically decreased to less than 30%. When the ADH expression levels were lowered, most of the cyclohexanol was oxidized with ε-caprolactone yields of 71.8 and 87.0% for BDT-2 and BDR-2, respectively. Although the strain BDR-2 achieved the highest ε-caprolactone concentration as 34.8 mM, small amounts of cyclohexanol and cyclohexanone were still detected in the reaction mixture. The results indicate that the further tuning in the bi-enzyme tandem system is needed to improve the performance, especially at high concentrations of cyclohexanol substrate.

The activity ratio of ADH/CHMO is an important factor for the whole-cell synthesis of ε-caprolactone in the bi-enzymatic cascade biotransformation considering the NADP(H) balance. The highest ε-caprolactone yield was achieved at a ratio of ADH/CHMO of 0.34 for the strain BDR-2. For the starting strain of BDT-1 and BDR-1, the ratios of ADH/CHMO were in the range of 0.06–0.07. In the bi-enzymatic system, 1 mol of NADPH is produced through the ADH catalysis from cyclohexanol, accompanying 1 mol of NADPH consumed by CHMO to produce 1 mol of ε-caprolactone (Fig. 1). From the stoichiometry perspective, the closer to 1 the ratio of ADH/CHMO is, the better the ε-caprolactone biosynthesis efficiency in the cascade reaction. Thus, it was speculated that the regulation of CHMO expression level might be beneficial to improve the ε-caprolactone activities in this tandem cascade catalytic reaction.

### CHMO expression level tuning

**Fig. 4:**
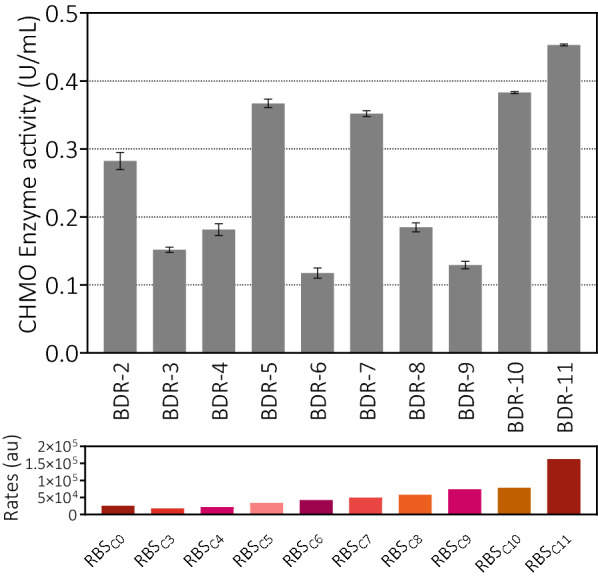
CHMO activities in *E. coli* crude cell lysates which harbored different RBS sequences for CHMO co-expressed with ADH in the pRSFDuet vectors. Enzyme activities were determined by the NADPH assay using cyclohexanone as substrate. The translation initiation rates calculated from the RBS Calculator V2.0 (Espah Borujeni et al. 2014) were presented below the activities. The standard deviations were derived from three independent experiments

In this study, a series of RBS sequences controlling CHMO expression were designed with different T7 RNAP translation rates according to the RBS Calculator V2.0 (Additional file 1: Table S3) (Espah Borujeni et al. 2014). After the replacements of RBS sequences, there was little difference in the ADH activity of different recombinant cells as expected. However, the CHMO activities were altered dramatically (Fig. 4). Generally, the expression levels of CHMO judged from SDS-PAGE were roughly consistent with the detected CHMO activities. And the expression levels of ADH changed insignificantly (Additional file 1: Fig. S2). When the translation rates were reduced, the activities of CHMO also decreased to different degrees (RBS_C3_ and RBS_C4_). For the sequence replacements of RBS_C5_, RBS_C7_, RBS_C10_ and RBS_C11_ with faster RNAP translation rate, activities of CHMO were elevated by 25–60% compared with that of RBS_C0_. But a faster translation rate did not always indicate a relatively higher CHMO activities (e.g., RBS_C6_, RBS_C8_ and RBS_C9_).

**Fig. 5:**
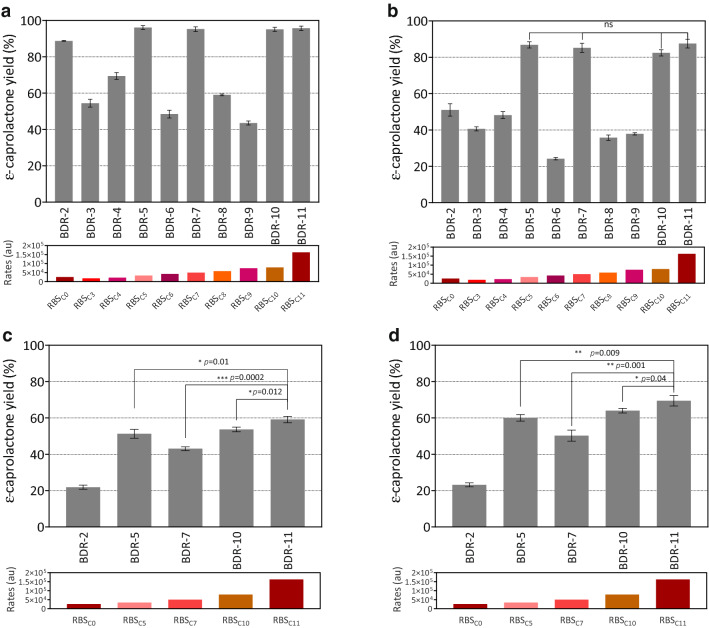
Effect of different translate rates of RBSs on ε-caprolactone yields catalyzed by the *E. coli* BL21 (DE3) cells using 40 mM (**a**), 60 mM (**b**), 80 mM (**c** & **d**) cyclohexanol as substrates. The whole-cell biocatalysis was carried out in triplicate at 25 ℃ for 16 h (**a**, **b** and **c**) and 24 h (**d**). Data were expressed as the mean values accompanied by the standard deviations. The significant analysis between the data was performed through multiple t tests by GraphPad Prism 8.0. *, *p* < 0.05, **, *p* < 0.01, ***, *p* < 0.001, ns, not significant

The engineered strains containing different expression vector had no limiting effects on the growth behavior. But the whole-cell catalytic activity for ε-caprolactone biosynthesis of cells containing different RBS sequences showed great differences. Generally speaking, the cells with higher CHMO enzyme activities presented better catalytic performance (Fig. 5 and Additional file 1: Fig. S3). When the RBS_C0_ was replaced by the RBS_C5_, RBS_C7_, RBS_C10_, or RBS_C11_, the ε-caprolactone yields were improved to about 95.5 ± 0.4% using 40 mM cyclohexanol as substrate. With the enhancement of substrate concentration, the values of ε-caprolactone yield were reduced and the differences between them were gradually more significant. For the starter strain of BDR-2 with RBS_C0_ controlling CHMO expression, the ε-caprolactone yield was only about 22% with 80 mM cyclohexanol as substrate after 16 h catalysis. For the other four optimized strains, the ε-caprolactone yields were in the range from 43.1 to 59.2%. When the reaction time extended to 24 h, the ε-caprolactone yields were further increased to 50.2–69.5%. A dramatic decrease of conversion of cyclohexanol to ε-caprolactone was recorded at 60 mM substrate concentration. This might be attributed to the inhibition by product or substrate to the CHMO (Kohl et al. 2018).

Above results show that the strain BDR-11 presented the best ε-caprolactone synthesis capability, which was equipped with RBS_C11_ at the putative T7 RNAP translation rate of 162,491.50 au. This RBS change from RBS_C0_ caused the activity ratio of CHMO/ADH to rise from 0.34 to 0.66. The relative balance of enzyme activities promoted the balance between the supply and consumption of NADP(H), thereby improving the tolerance of the cascade catalytic system to the substrate and the product conversion.

The ratios of NADPH/NADP^+^ were determined in the strain BDR-2 and BDR-11 (Additional file 1: Table S5). In the initial stage of the whole-cell catalysis reaction, the ratios of NADPH/NADP^+^ of the two strains were similar. After 16 h of catalytic reaction, a great change of cellular NADPH/NADP^+^ ratio in BDR-2 was observed (from 0.105 to 0.138). For the strain BDR-11, the change of cytosolic NADPH/NADP^+^ ratio was not significant. The results implied that BDR-11 displayed the better balance between the cofactor production and consumption.

### Production of ε-caprolactone

**Fig. 6:**
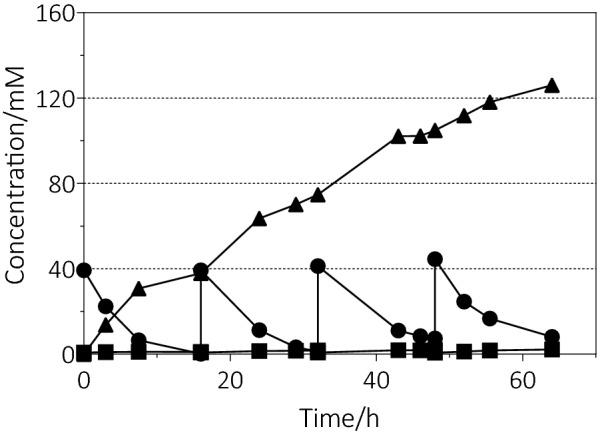
Fed-batch production of ε-caprolactone through the whole-cell catalysis with resting cells. 40 mM cyclohexanol was added into the reaction system every 16 h. 0.5-mL samples were taken out for the concentration determination of cyclohexanol (black circle), cyclohexanone (black square) and ε-caprolactone (black triangle)

As mentioned above, a substrate inhibition was found involved in the whole-cell bioconversion for ε-caprolactone production. The whole-cell biocatalysis was restricted by a high initial substrate concentration, which generally increases the process time and cost. To solve the problem, the production performance of the engineered strain BDR-11 was evaluated by whole-cell biocatalysis with a fed-batch strategy using resting cells. Batch reactions were carried out to produce ε-caprolactone by replacing 40 mM cyclohexanol as substrate three times. As shown in Fig. 6, cyclohexanol was almost completely consumed to ε-caprolactone with a small amount of cyclohexanone detected in the first two reaction batches. The concentration of ε-caprolactone increased to approximately 37.9 and 74.7 mM, respectively. With increasing reaction time and the numbers of feedings, the biocatalytic abilities of the BDR-11 declined gradually. 7.4 mM of cyclohexanol was detected with an 88% ε-caprolactone yield at the end of third reaction. The remaining cyclohexanol inhibited the further catalytic efficiency of the cells. After four batch reactions, the final concentration of ε-caprolactone reached at 126.0 mM with a total yield of 78.8%. To further evaluate the production performance of the engineered strain BDR-11, 60 mM cyclohexanol was used in fed-batch catalysis subsequently. But the high concentration substrate may inhibit the activities of CHMO and ADH, the conversion of cyclohexanol to ε-caprolactone was not high (Additional file 1: Fig. S4).

In previous studies, bioconversion for ε-caprolactone was reported using recombinant *Pseudomonas taiwanensis* (Karande et al. 2018), *Geotrichum candidum* (Silva et al. 2017) and *E. coli* cells by fed-batch (Kohl et al. 2018; Lee et al. 2007; Schmidt et al. 2015b) (Table 1). Among them, the maximum ε-caprolactone concentration of 134 mM was obtained using cyclohexanone (245 mM) as substrate by co-expression of glucose-6-phosphate dehydrogenase at a high cell density (35 g/L dried cells) (Lee et al. 2007). The recorded final conversion rate was about 54.7 mol/mol cyclohexanone. In this study, only 10 g/L wet cells achieved the catalytic process of converting 160 mM cyclohexanol into 126.0 mM ε-caprolactone. To our best knowledge, this is the highest catalytic efficiency for producing ε-caprolactone in the whole-cell batch reactions.

**Table 1:** Production of ε-caprolactone in different engineering host strains

Cells		Substrate		ε-caprolactone	Reaction	Yield (mol/mol substrate/cell mass)	References
Type	Mass (g/L)	Type	Concentration (mM)	Concentration (mM)			
*Pseudomonas taiwanensis*	6.8^a^	Cyclohexane	200	17.0	Single batch	0.0125 ^a^	(Karande et al. 2018)
*Geotrichum candidum*	100	Cyclohexanol	10	10	Single batch	0.0100	(Silva et al. 2017)
			60	58.4	Six batches	0.0097	
*E. coli*	10	Cyclohexanol	60 100	36^c^ 28^c^	Single batch	0.0600 0.0280	(Kohl et al. 2018)
*E. coli*	100	Cyclohexanol ^b^	60	57^c^	Single batch	0.0095	(Schmidt et al. 2015b)
			80	35^c^	Single batch	0.0044	
*E. coli*	35^a^	Cyclohexanone	245^c^	134	Four batches	0.0156 ^a^	(Lee et al. 2007)
*E. coli*	10	Cyclohexanol	60	47.7	Single batch	0.0795	This study
			80	50.5	Single batch	0.0631	
			160	126.0	Four batches	0.0788	

^a^Dry cell mass concentration

^b^Reaction mixtures were supplemented with equimolar amount of D-glucose and/or acetone for cofactor recycling

^c^Calculated from figures in the literature

## Conclusions

In this study, a route using whole-cell biocatalysis was designed to produce ε-caprolactone by cyclohexanol. With the help of RBS engineering, the expression levels of ADH and CHMO were fine-tuned to increase the operational efficiency. After optimization, an increase in the substrate tolerance was observed from 20 to 60 mM in single batch catalysis. Finally, the high production of ε-caprolactone was achieved through fed-batch strategy. The construction of the strain (BDR-11) provides the possibility of industrial production of ε-caprolactone, and highlights the importance of controlling the cofactor of NADPH regeneration.

### Supplementary Information

**Additional file 1: Fig. S1.** Analysis and identification of cyclohexanol, cyclohexanone and ε-caprolactone. (A) GC-FID chromatogram pattern of standards. Retention times of acetophenone, cyclohexanol, cyclohexanone and ε-caprolactone were 7.444, 7.648, 12.158 and 10.745 min, respectively. Mass spectra of cyclohexanol (B), cyclohexanone (C) and ε-caprolactone (D) were compared with the authentic standards. **Fig. S2.** SDS-PAGE analysis of recombinant E. coli cells when the RBS sequences controlling CHMO expression on pRSFDuet-1 were altered. Strains from BDR-02 to BDR-11 were abbreviated from 02 to 11. **Fig. S3.** Effects of different RBS sequences on the production of ε-caprolactone using different concentrations of cyclohexanol as substrates catalyzed for different hours. (A) 40 mM, 16 h; (B) 60 mM, 16 h; (C) 80 mM, 16 h; (D) 80 mM, 24 h. The calculated translation rates were also presented. Three independent experiments were performed. **Fig. S4.** Fed-batch production of ε-caprolactone through the whole-cell biocatalysis. 60 mM cyclohexanol was added into the reaction system every 20 h. 0.5 mL samples were taken out for the concentration determination of cyclohexanol (Black circle), cyclohexanone (Black square) and ε-caprolactone (Black triangle). **Table S1.** Recombinant E. coli BL21(DE3) strains and plasmids used in this study. **Table S2.** Primers used in this study. **Table S3.** The designed ribosome binding sites of CHMO and ADH genes and their predicted translation initiation rates. **Table S4.** The measured final OD600 of the engineered strains before centrifugation for harvest. All experiments were performed in triplicates. **Table S5.** Changes of cellular NADPH/NADP+ ratios in BDR-2 and BDR-11*.

## Data Availability

The datasets used and/or analyzed during the current study are available from the corresponding author on reasonable request.

## Abbreviations

ADH: Alcohol dehydrogenase
CHMO: Cyclohexanone monooxygenase
NADH: Nicotinamide adenine dinucleotide
NADPH: Nicotinamide adenine dinucleotide phosphate
RBS: Ribosome binding site
IPGT: Isopropyl β-D-1-thiogalactopyranoside
SDS-PAGE: Sodium dodecyl sulfate-polyacrylamide gel electrophoresis
DMSO: Dimethyl sulfoxide
LB: Luria–Bertani medium
WCW: Wet cell weight
GC: Gas chromatography
FID: Flame-ionization detector
GC–MS: Chromatography coupled to mass spectrometry
MSD: Mass selective detector
