# Supplementary material for: Tuning a bi-enzymatic cascade reaction in Escherichia coli to facilitate NADPH regeneration for ε-caprolactone production
Source: Bioresour Bioprocess. 2021 Apr 22;8(1):32. doi: 10.1186/s40643-021-00370-w (PMC10992311; doi:10.1186/s40643-021-00370-w)
Supplement: Supplementary file 1 — Additional file 1: Fig. S1. Analysis and identification of cyclohexanol, cyclohexanone and ε-caprolactone. (A) GC-FID chromatogram pattern of standards. Retention times of acetophenone, cyclohexanol, cyclohexanone and ε-caprolactone were 7.444, 7.648, 12.158 and 10.745 min, respectively. Mass spectra of cyclohexanol (B), cyclohexanone (C) and ε-caprolactone (D) were compared with the authentic standards. Fig. S2. SDS-PAGE analysis of recombinant E. coli cells when the RBS sequences controlling CHMO expression on pRSFDuet-1 were altered. Strains from BDR-02 to BDR-11 were abbreviated from 02 to 11. Fig. S3. Effects of different RBS sequences on the production of ε-caprolactone using different concentrations of cyclohexanol as substrates catalyzed for different hours. (A) 40 mM, 16 h; (B) 60 mM, 16 h; (C) 80 mM, 16 h; (D) 80 mM, 24 h. The calculated translation rates were also presented. Three independent experiments were performed. Fig. S4. Fed-batch production of ε-caprolactone through the whole-cell biocatalysis. 60 mM cyclohexanol was added into the reaction system every 20 h. 0.5 mL samples were taken out for the concentration determination of cyclohexanol (Black circle), cyclohexanone (Black square) and ε-caprolactone (Black triangle). Table S1. Recombinant E. coli BL21(DE3) strains and plasmids used in this study. Table S2. Primers used in this study. Table S3. The designed ribosome binding sites of CHMO and ADH genes and their predicted translation initiation rates. Table S4. The measured final OD600 of the engineered strains before centrifugation for harvest. All experiments were performed in triplicates. Table S5. Changes of cellular NADPH/NADP+ ratios in BDR-2 and BDR-11*. [file 40643_2021_370_MOESM1_ESM.docx]

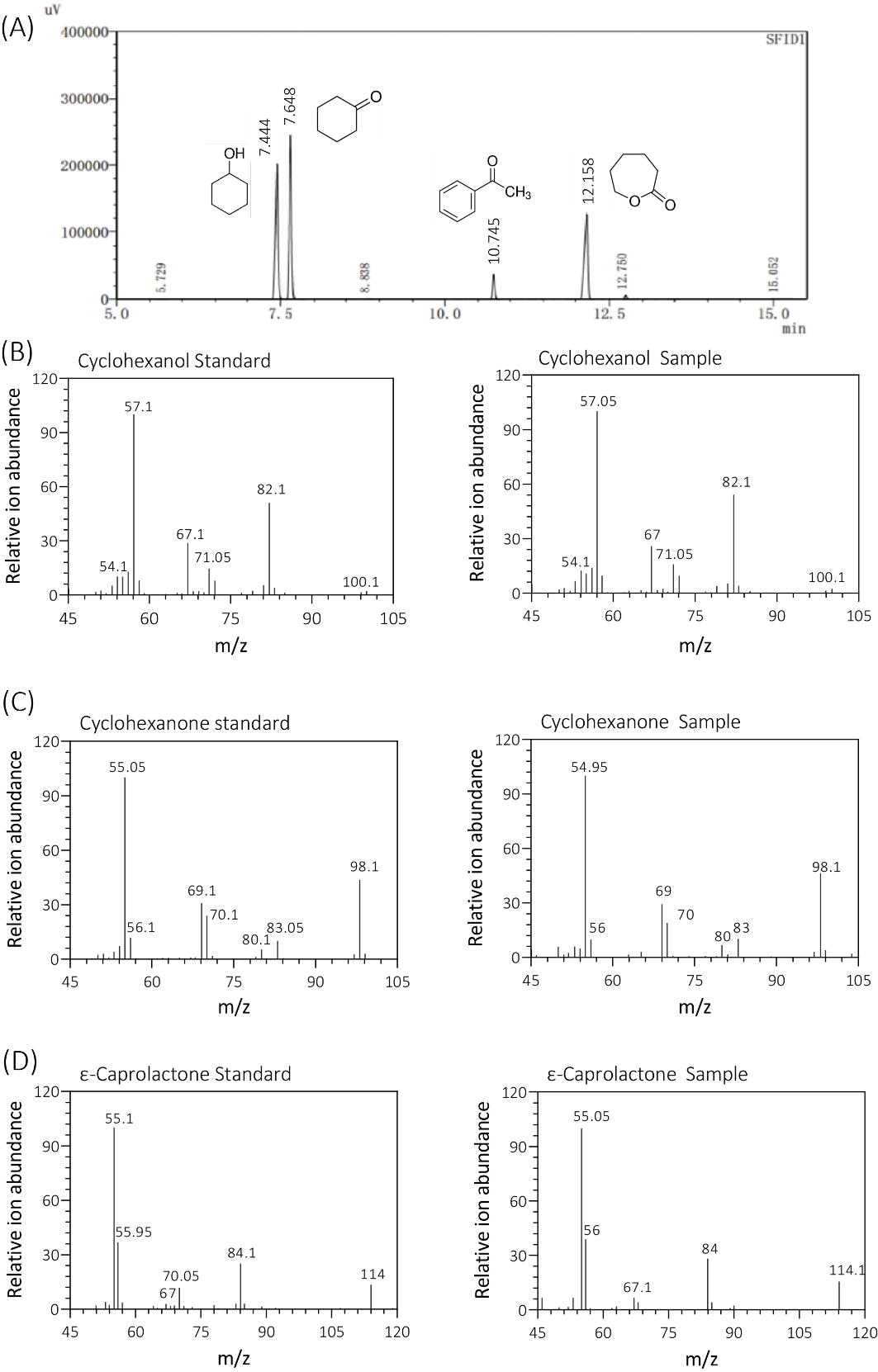


# **Fig. S1** Analysis and identification of cyclohexanol, cyclohexanone and ε-caprolactone. (A) GC-FID chromatogram pattern of standards. Retention times of acetophenone, cyclohexanol, cyclohexanone and ε-caprolactone were 7.444, 7.648, 12.158 and 10.745 min, respectively. Mass spectra of cyclohexanol (B), cyclohexanone (C) and ε-caprolactone (D) were compared with the authentic standards.


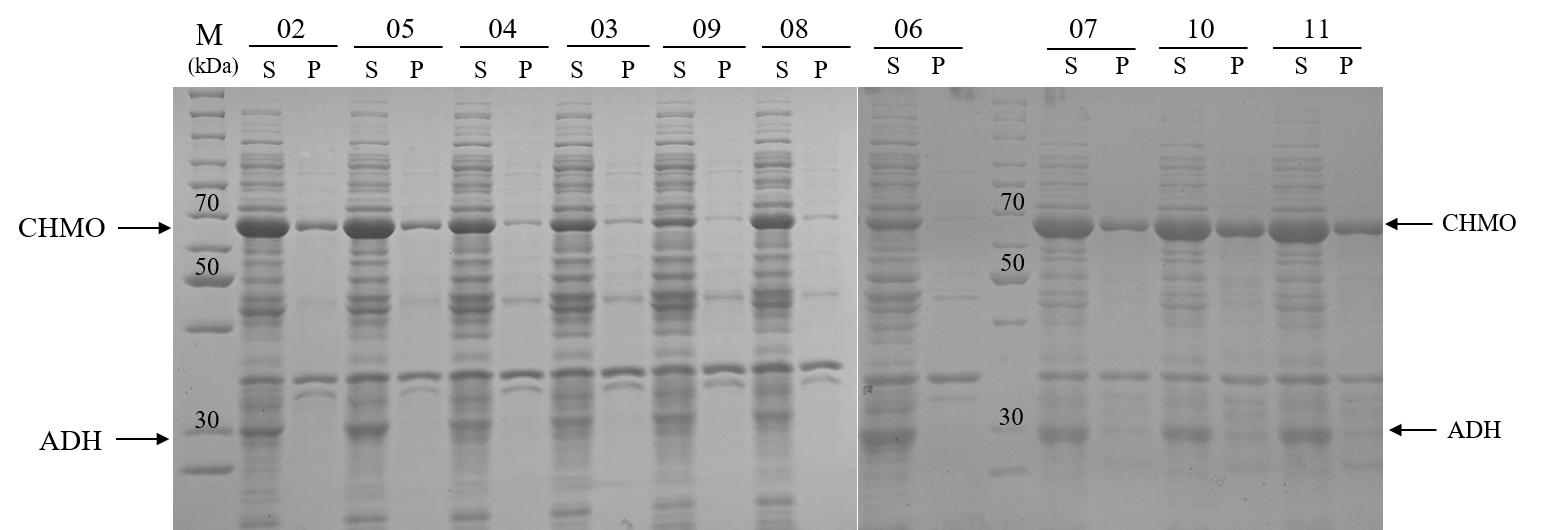


# **Fig. S2** SDS-PAGE analysis of recombinant *E. coli* cells when the RBS sequences controlling CHMO expression on pRSFDuet-1 were altered. Strains from BDR-02 to BDR-11 were abbreviated from 02 to 11.


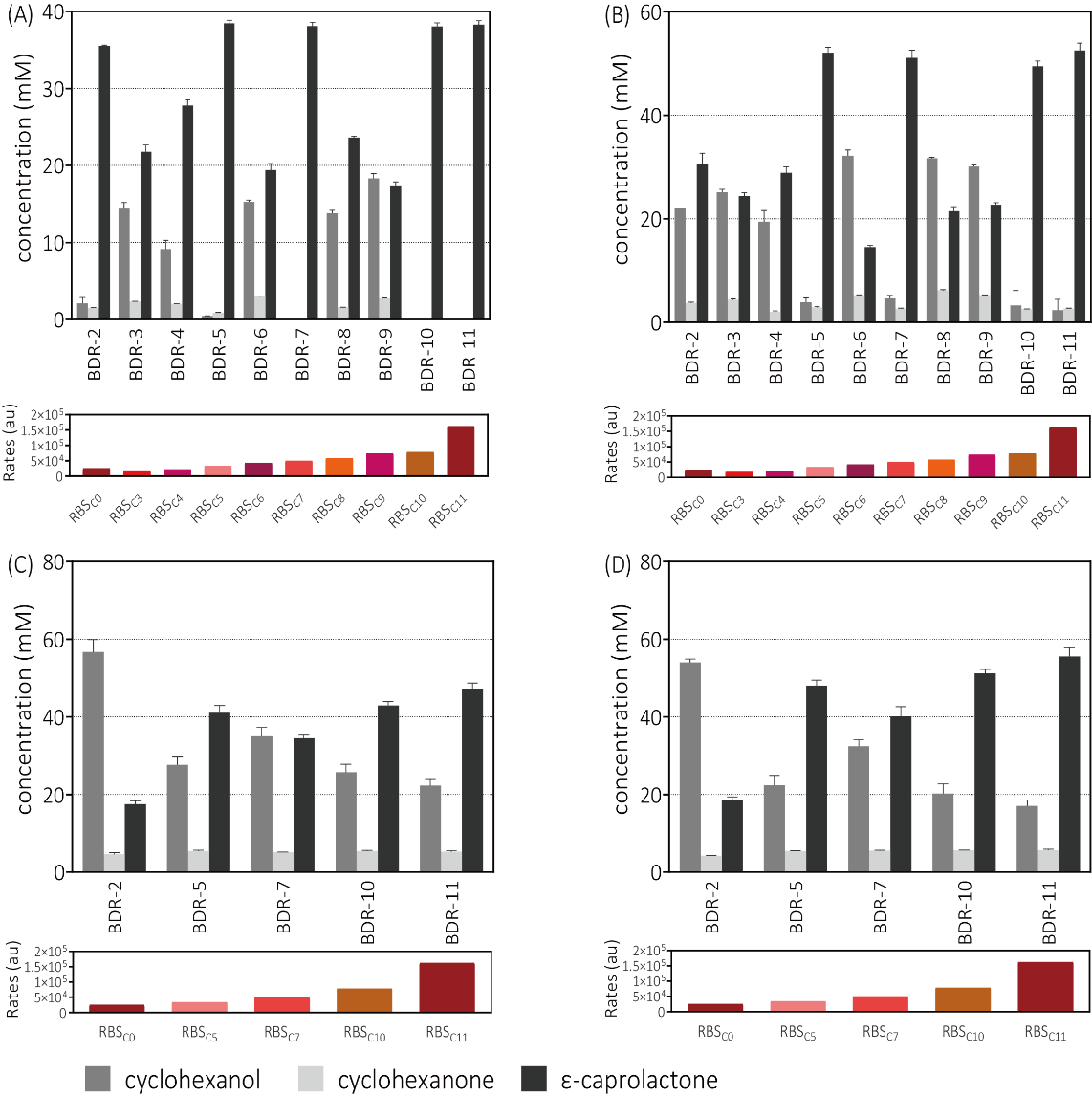


# **Fig. S3** Effects of different RBS sequences on the production of ε-caprolactone using different concentrations of cyclohexanol as substrates catalyzed for different hours. (A) 40 mM, 16 h; (B) 60 mM, 16 h; (C) 80 mM, 16 h; (D) 80 mM, 24 h. The calculated translation rates were also presented. Three independent experiments were performed.

# **Fig. S4** Fed-batch production of ε-caprolactone through the whole-cell biocatalysis. 60 mM cyclohexanol was added into the reaction system every 20 h. 0.5 mL samples were taken out for the concentration determination of cyclohexanol (●), cyclohexanone (■) and ε-caprolactone (▲).

**Table S1** Recombinant *E. coli* BL21(DE3) strains and plasmids used in this study.

| Strains | Vector | RBS for CHMO | RBS for ADH |
| --- | --- | --- | --- |
|  | pETD-CHMO | RBS_C0_ | - |
|  | pETD-ADH | - | RBS_A0_ |
| BDT-1 | pET-C0A0 | RBS_C0_ | RBS_A0_ |
| BDT-2 | pET-C0A1 | RBS_C0_ | RBS_A1_ |
| BDR-1 | pRSF-C0A0 | RBS_C0_ | RBS_A0_ |
| BDR-2 | pRSF-C0A1 | RBS_C0_ | RBS_A1_ |
| BDR-3 | pRSF-C3A1 | RBS_C3_ | RBS_A1_ |
| BDR-4 | pRSF-C4A1 | RBS_C4_ | RBS_A1_ |
| BDR-5 | pRSF-C11A1 | RBS_C5_ | RBS_A1_ |
| BDR-6 | pRSF-C12A1 | RBS_C6_ | RBS_A1_ |
| BDR-7 | pRSF-C13A1 | RBS_C7_ | RBS_A1_ |
| BDR-8 | pRSF-C14A1 | RBS_C8_ | RBS_A1_ |
| BDR-9 | pRSF-C15A1 | RBS_C9_ | RBS_A1_ |
| BDR-10 | pRSF-C16A1 | RBS_C10_ | RBS_A1_ |
| BDR-11 | pRSF-C17A1 | RBS_C11_ | RBS_A1_ |

**Table S2** Primers used in this study.

| Primers | Sequences (5′-3′) | Description |
| --- | --- | --- |
| CHMO-f | TTTACACCATGGGCAGCAGCCATCA | *chmo* gene cloning |
| CHMO-r | TTTACACTGCAGTTATGCGTTAGCC |  |
| ADH-f | CCCCATCTTAGTATATTAGTTA | *adh* gene with RBS_A1_ cloning |
| ADH-r | AGCAGCGGTTTCTTTACCAGACTCGAGTTACTGCGCGGTG |  |
| pD-f | ACTAATATACTAAGATGGGGAATTGTTA | The backbones of pRSFDuet |
| pD-r | TCTGGTAAAGAAACCGCTGCTGCGAAA |  |
| pD1-f | CTGCAGGTCGACAAGCTTGCGGCCGCATAATGCTTA | The backbones of pRSF-C0A0 and pRSF-C0A1 cloning |
| pD1-r | GGTATATCTCCTTATTAAAGTTAAACAAAATTAT |  |
| pD2-f | CTCGAGTCTGGTAAAGAAACCGCTGCTGCGAAATTTGAACGC |  |
| pD2-r | ATGTATATCTCCTTCTTATACTTAACTAATATACTAAG |  |
| ApD1-f | CTTTAATAAGGAGATATACCATGACGGATCGTCTGAAAGG |  |
| ApD1-r | GCAAGCTTGTCGACCTGCAGTTACTGCGCGGTGTAACCACCATCAACAA |  |
| CpD2-f | TATAAGAAGGAGATATACATATGGGCAGCAGCCATCACCATCAT | RBS_C0_-CHMO fragment cloning |
| CpD2-r | GTTTCTTTACCAGACTCGAGTTATGCGTTAGCCGGCTGTTTAATAT |  |
| CR-f | ATTTTTGAAAAAGCCGTTTCTGTAAT | The backbone of pRSF-C0A1 cloning |
| CR-r | GCTTTTTCAAAAATATGGTATTGATA |  |
| RBS_C3_-f | CTATTCTATCATTAAGGAGGAGTCCCATGGGCAGCAGCCATCAC | RBS_C3_ fragment cloning |
| RBS_C3_-r | GACTCCTCCTTAATGATAGAATAGATTAAAGTTAAACAAAATTA |  |
| RBS_C4_-f | TCGAAATACTAAGGAGACTTTTTCCATGGGCAGCAGCCATCACCA | RBS_C4_ fragment cloning |
| RBS_C4_-r | AAAAAGTCTCCTTAGTATTTCGAATTAAAGTTAAACAAAATTA |  |
| RBS_C5_-f | ACACAACATTTAATAAGGAGCTTTTTTCCATGGGCAGCAGCCATCA | RBS_C5_ fragment cloning |
| RBS_C5_-r | AAAAAAGCTCCTTATTAAATGTTGTGTATTAAAGTTAAACAAAATTA |  |
| RBS_C6_-f | ACTAAACCCTTCTTTAAGGAGGTAATACCCATGGGCAGCAGCCATCAC | RBS_C6_ fragment cloning |
| RBS_C6_-r | GTATTACCTCCTTAAAGAAGGGTTTAGTATTAAAGTTAAACAAAATTA |  |
| RBS_C7_-f | CAGCAAAAGTAAGGAGGTATTCTTCCATGGGCAGCAGCCATCA | RBS_C7_ fragment cloning |
| RBS_C7_-r | AAGAATACCTCCTTACTTTTGCTGATTAAAGTTAAACAAAATTA |  |
| RBS_C8_-f | TGTTTAAAGTACCTAAGGAACCTTTTATGGGCAGCAGCCATCA | RBS_C8_ fragment cloning |
| RBS_C8_-r | AAAAGGTTCCTTAGGTACTTTAAACAATTAAAGTTAAACAAAATTA |  |
| RBS_C9_-f | CTATTCTATCATTAAGGAGGAGTCATGGGCAGCAGCCATCAC | RBS_C9_ fragment cloning |
| RBS_C9_-r | GACTCCTCCTTAATGATAGAATAGATTAAAGTTAAACAAAATT |  |
| RBS_C10_-f | CGATCAAAACCTAAGGAGACCTTTATGGGCAGCAGCCATCACCATCAT | RBS_C10_ fragment cloning |
| RBS_C10_-r | AAAGGTCTCCTTAGGTTTTGATCGATTAAAGTTAAACAAAATTA |  |
| RBS_C11_-f | GAAAAAGTCCTAAGGAGGAAATTTATGGGCAGCAGCCATCACCATCATC | RBS_C11_ fragment cloning |
| RBS_C11_-r | AAATTTCCTCCTTAGGACTTTTTCATTAAAGTTAAACAAAATTA |  |

The underline in red indicated the sequence of the synthetic RBS.

**Table S3** The designed ribosome binding sites of CHMO and ADH genes and their predicted translation initiation rates.

| RBS | Sequence | Translation initiation rates /au | Accuracy (warnings) |
| --- | --- | --- | --- |
| RBS_A0_ | AAGGAGATATACAT | 2,020.17 | NoEQ |
| RBS_A1_ | AACGAGATATACAT | 343.01 | NoEQ |
| RBS_C0_ | AAGGAGATATACC | 25,441.85 | NoEQ |
| RBS_C3_ | ACACAACATTTAATAAGGAGCTTTTTTCC | 18,569.94 | OK |
| RBS_C4_ | TCGAAATACTAAGGAGACTTTTTCC | 22,132.81 | OK |
| RBS_C5_ | CTATTCTATCATTAAGGAGGAGTCCC | 34,281.13 | NoEQ |
| RBS_C6_ | TGTTTAAAGTACCTAAGGAACCTTTT | 42,723.36 | OK |
| RBS_C7_ | CTATTCTATCATTAAGGAGGAGTC | 50,224.51 | OK |
| RBS_C8_ | CAGCAAAAGTAAGGAGGTATTCTTCC | 58,276.47 | OK |
| RBS_C9_ | ACTAAACCCTTCTTTAAGGAGGTAATACCC | 74,465.67 | OK |
| RBS_C10_ | CGATCAAAACCTAAGGAGACCTTT | 78,490.81 | OK |
| RBS_C11_ | GAAAAAGTCCTAAGGAGGAAATTT | 162,491.5 | OK |

# Table S4 The measured final OD_600_ of the engineered strains before centrifugation for harvest. All experiments were performed in triplicates.

| Strains | OD_600_ | | | | |
| --- | --- | --- | --- | --- | --- |
|  | 1 | 2 | 3 | Average | SD |
| BDT-1 | 2.420 | 2.504 | 2.535 | 2.486 | 0.060 |
| BDT-2 | 2.308 | 2.364 | 2.300 | 2.324 | 0.035 |
| BDR-1 | 2.504 | 2.496 | 2.314 | 2.438 | 0.107 |
| BDR-2 | 2.608 | 2.260 | 2.466 | 2.445 | 0.175 |

# **Table S5** Changes of cellular NADPH/NADP^+^ ratios in BDR-2 and BDR-11^*^

| Strain | 0 h | 16 h |
| --- | --- | --- |
| BDR-2 | 0.105±0.002 | 0.138±0.009 |
| BDR-11 | 0.103±0.001 | 0.105±0.004 |

^*^ Ratios of cellular NDAPH/NADP^+^ were determined when the whole-cell catalyzed ε-caprolactone production using 60 mM of cyclohexanol as substrate at 0 h and 16 h. Experiments were performed in three independent samples.
